# Sustained-releasing hollow microparticles with dual-anticancer drugs elicit greater shrinkage of tumor spheroids

**DOI:** 10.18632/oncotarget.20591

**Published:** 2017-08-24

**Authors:** Jong-Suep Baek, Chee Chong Choo, Nguan Soon Tan, Say Chye Joachim Loo

**Affiliations:** ^1^ School of Materials Science and Engineering, Nanyang Technological University, 639798, Singapore; ^2^ School of Biological Sciences, Nanyang Technological University, 637551, Singapore; ^3^ Lee Kong Chian School of Medicine, Nanyang Technological University, 639798, Singapore; ^4^ Institute of Molecular Cell Biology, Proteos, Agency for Science Technology and Research, 138673, Singapore; ^5^ KK Research Centre, KK Women's and Children Hospital, 229899, Singapore; ^6^ Singapore Centre on Environmental Life Sciences Engineering, Nanyang Technological University, 637551, Singapore

**Keywords:** microparticles, controlled-release, PLGA, combination therapy, paclitaxel

## Abstract

Polymeric particulate delivery systems are vastly explored for the delivery of chemotherapeutic agents. However, the preparation of polymeric particulate systems with the capability of providing sustained release of two or more drugs is still a challenge. Herein, poly (D, L-lactic-co-glycolic acid, 50:50) hollow microparticles co-loaded with doxorubicin and paclitaxel were developed through double-emulsion solvent evaporation technique. Hollow microparticles were formed through the addition of an osmolyte into the fabrication process. The benefits of hollow over solid microparticles were found to be higher encapsulation efficiency and a more rapid drug release rate. Further modification of the hollow microparticles was accomplished through the introduction of methyl-β-cyclodextrin. With this, a higher encapsulation efficiency of both drugs and an enhanced cumulative release were achieved. Spheroid study further demonstrated that the controlled release of the drugs from the methyl-β-cyclodextrin -loaded hollow microparticles exhibited enhanced tumor regressions of MCF-7 tumor spheroids. Such hollow dual-drug-loaded hollow microparticles with sustained releasing capabilities may have a potential for future applications in cancer therapy.

## INTRODUCTION

Globally, cancer remains one of the leading cause of human mortality. Based on a report by IMS Health in 2016, the global market for cancer therapy, at an annual growth rate of 7.5 – 10.5 %, is expected to reach $150 billion by 2020 [1]. Today, the main treatment for cancer is tumor removal through surgical means, but in situations where the malignant cells are no longer localized, chemotherapy will be the principal treatment modality. Under such a circumstance, and if the situation permits, combination chemotherapy is desired. Combination chemotherapy aims to achieve a more efficacious treatment [2–5], whereby drugs of different mechanisms of action are used in combination, to achieve a synergistic advantage while minimizing their dreadful side effects. For example, several clinical studies have reported that the co-delivery of doxorubicin (DOX) and paclitaxel (PTX) increases tumor regression rates as compared to the use of a single drug [6–7]. In addition, a combinatory drug approach also curbs the drug-resistant evolution of tumors [8].

With combination therapy, the co-delivery of different chemotherapeutic drugs, through the use of particulate carriers as a delivery system is, therefore, an attractive strategy. This is especially so when sustained delivery of the drugs is required. Biocompatible and biodegradable polyesters, developed into particulate drug delivery systems, are therefore excellent material candidates for the encapsulation of these highly sensitive yet cytotoxic drugs while providing an added functionality of controlled release. In fact, encapsulating drugs into microparticles that provide a continuous release of a single anticancer drug has already been shown to inhibit tumor growth [9, 10]. Some commercially available single-drug delivery systems include Doxil®, Caelyx® and Myocet® – liposomal-based systems for DOX delivery, but they suffer from drug leakage and particle aggregation in these formulations [11]. For the delivery of PTX, Taxol® – a formulation with Cremophor EL, is used but not without the severe side effects experienced by patients [12]. Abraxane® – a protein-bound paclitaxel formulation does to a certain extent resolve this issue but a sustained delivery formulation is currently unavailable. As such, combination chemotherapy, therefore requires the patient to undergo multiple drug administrations from single-drug formulations in order to reap their synergistic benefits [13]. In addition, these commercially available formulations also lack the slow continuous release that is often highly desirable. With the advent of combination therapy, a whole new approach in developing delivery systems that deliver multiple drugs in a sustained manner is now required.

While myriad of delivery systems have been developed, microparticulate systems for drug delivery as reported in the scientific literature have their limitations, and achieving controlled release of more than one drug is always a challenge. For example, recent papers that report on multiple drug encapsulation do not focus on achieving controlled release [14, 15]. While a few studies report on the co-delivery of two anticancer drugs from a single particulate formulation [16, 17], the release profiles of these multiple drugs cannot be easily adjusted. Achieving controlled release in combination therapy is critical because the likelihood of severe side effects with the use of multiple drugs is higher compared to the administration of a single drug [18, 19]. Another issue with co-drug delivery lies in the ability to overcome poor drug encapsulation efficiency (EE), especially hydrophilic drugs, within a single formulation [20]. This is another key consideration as it would strongly influence the cost effectiveness of pharmaceutical reformulations.

The aim of this work was therefore to co-deliver two anticancer drugs, i.e. DOX and PTX, from hollow microparticles and compare its efficacy against single-drug-loaded microparticles in tumor spheroids. Hollow particles are preferred over solid particles because the former uses less polymer per particle, and our earlier studies showed that they allow for a complete release of the encapsulated drug [21]. This maximizes on a higher drug-to-polymer (w/w %) ratio as compared to solid microparticles. In addition, we further investigated the co-encapsulation of methyl-β-*cyclodextrin* (*MCD*) into hollow microparticles can enhance the toxic effect of DOX. We hypothesized that drug-loaded hollow microparticles, with *MCD*, would achieve better tumor shrinkage outcomes in spheroid studies. DOX/MCD inclusion complex is known to exhibit pro-apoptotic function because of the activation of the extrinsic apoptotic pathway via p53 [22]. Here, human breast cancer cells (i.e. MCF-7) was challenged against these drug-loaded microparticles to investigate for their tumor regression efficacy.

## RESULTS AND DISCUSSION

### Drug-loaded hollow PLGA microparticles without MCD

**Figure 1:**
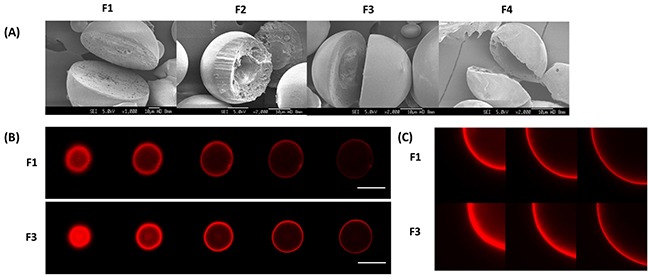
(A) SEM images of solid microparticle (F1) and hollow microparticles (F2-F4) **(B)** z-stack comprising five confocal sections was obtained for DOX (red) of F1 and F3. Scale bar = 30 μm. **(C)** z-stack comprising three zoomed-in confcoal sections of F1 and F3.

Solid and hollow PLGA microparticles were fabricated by the double emulsion solvent evaporation method. Figure 1A shows the SEM images of these microparticles that had earlier been excised to reveal their inner structures. The particle size of solid PLGA microparticles (F1), as measured by the SEM, was 31.5 ± 9.7 μm, which is similar to that of the hollow formulations (32.1 ∼ 37.6 μm). This size range is suitable for the particles to be employed as drug depot systems [23, 24], as they can be localized at the site of injection to provide sustained drug release [25]. Hollow PLGA microparticles were fabricated using NaCl, of varying amounts (Table 1). During freeze drying, as the water content in the core was removed, microparticles containing a hollow cavity were generated. By altering the amount of NaCl, microparticles of different cavity sizes and shell thicknesses were obtained, and this is largely driven by the osmotic pressure achieved from the salt. Higher osmolyte concentration allows for more water influx into the emulsion droplet [21], thus translating to a larger cavity. The cavity diameters achieved were 9.3 ± 3.5 μm, 13.5 ± 4.2 μm and 20.3 ± 8.5 μm, for F2 (3 mg of NaCl), F3 (5 mg of NaCl) and F4 (10 mg of NaCl), respectively. Higher osmolyte content therefore drives larger volumes of water from the W_2_ aqueous phase into the emulsion droplet to generate microparticles with larger cavities.

**Table 1:** Encapsulation efficiency (%) of DOX and PTX in various microparticles (n=3, mean ±SD)

Samples	DOX	PTX
F1 (PLGA MP)	35.2 ± 3.9	88.6 ± 4.5
F2 (DOX+PTX-loaded PLGA hollow MP – 3 mg of NaCl)	44.7 ± 4.1	85.2 ± 4.5
F3 (DOX+PTX-loaded PLGA hollow MP – 5 mg of NaCl)	49.2 ± 2.6	83.7 ± 2.9
F4 (DOX+PTX-loaded PLGA hollow MP – 10 mg of NaCl)	27.6 ± 7.4	67.7 ± 3.7
F5 (DOX+MCD and PTX-loaded PLGA hollow MP – 5 mg of NaCl / 29.2 mg of MCD)	55.3 ± 4.8	82.1 ± 7.1
F6 (DOX+MCD and PTX-loaded PLGA hollow MP – 5 mg of NaCl / 58.8 mg of MCD)	68.7 ± 4.1	80.5 ± 5.7
F7 (DOX/MCD and PTX-loaded PLGA hollow MP – 5 mg of NaCl / 88.2 mg of MCD)	70.3 ± 5.7	70.1 ± 6.6

Next, by exploiting the intrinsic fluorescence of DOX, the localization of DOX within the drug-encapsulating microparticles was determined using CLSM. Figure 1B and 1C show CLSM images obtained for solid (F1) and hollow (F3) DOX-PTX-loaded PLGA microparticles. The red fluorescent rings, representative of DOX, were observed for samples F1 and F3, showing the location of DOX to be close to the particle surface. Next, the EE of DOX and PTX in these microparticles were measured (Table 1). PTX was found to have a higher EE in these microparticles compared to DOX. The hydrophobic nature of PTX tends to promote favorable interactions with hydrophobic PLGA [25]. As for hollow microparticles, they exhibited significantly higher EE of DOX as compared to the solid microparticles (F1). Notably, from the CLSM images, this higher EE of DOX is corroborated with a thicker red fluorescent ring of F3 as compared to F1. A higher osmolyte concentration likely increased water influx that correspondingly increased encapsulation of hydrophilic DOX.

**Figure 2:**
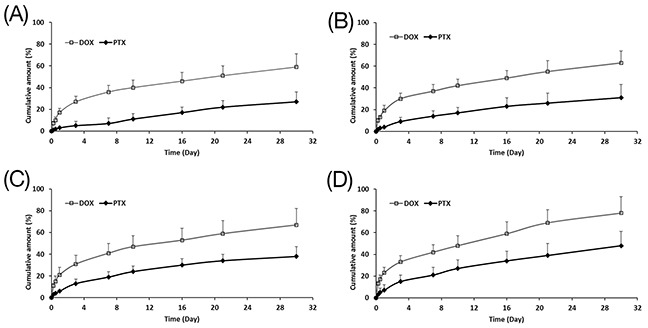
Cumulative release of DOX and PTX from **(A)** F1, **(B)** F2, **(C)** F3 and **(D)** F4 up to 30 days (n=3, mean ± S.D).

The drug release profiles from solid and hollow PLGA microparticles were subsequently investigated (Figure 2), and their corresponding release rates were tabulated in Table 2. For all microparticles, the release rate of DOX was noticeably faster than PTX because its hydrophilic nature promotes drug diffusion and solubility in the physiologically-relevant release medium. Comparing between different particle morphology, drug release rates for hollow microparticles (F2 to F4) were evidently faster as compared to solid microparticles (F1), and release rates also increased with increasing cavity size (Supplementary Figure 1). For particles of the same sizes, a larger cavity translates to a thinner shell, and this reduces the diffusion distance of the drug. While a shorter diffusion distance is one explanation, another reason for a more rapid release is the faster rate of hydrolysis for the hollow microparticles. Plotting the average molecular weight against time (Supplementary Figure 2A), hollow microparticles were shown to degrade faster than the solid microparticles, indicating that degradation rate is inversely correlated to shell thickness (i.e. cavity size). Microparticles with thinner shells promote water influx into the cavity of these microparticles. Together with a larger surface area of the internal cavity, polymer hydrolysis is therefore accelerated. In summary, larger cavity sizes or thinner-shelled particles result in faster drug release that is determined by both shorter diffusion distance and faster polymer degradation. Nevertheless, the cumulative release of both drugs is still low even up to 30 days. In view of this, the use of MCD was investigated to increase the cumulative release of both drugs within the 30-day period.

**Table 2:** Correlation coefficient (r^2^) and rate constant (*K*) of DOX and PTX from solid (F1) and hollow microparticles (F2-F7) after fitting to the Higuchi-equation

	Correlation coefficient r^2^		Rate constant *K* (h^−1^)	
	DOX	PTX	DOX	PTX
F1	0.9930	0.9422	1.6217	0.9264
F2	0.9750	0.9964	2.3307	1.1919
F3	0.9746	0.9978	2.5168	1.5624
F4	0.9835	0.9977	2.7595	1.7740
F5	0.9793	0.9886	3.0142	1.9544
F6	0.9851	0.9954	3.4512	2.1342
F7	0.9725	0.9928	3.9421	2.4218

### Drug-loaded hollow PLGA microparticles with MCD

Having established the influence of cavity size on drug release rates in hollow microparticles, *MCD* was next introduced into the hollow microparticles. MCD is reported to enhance the anti-tumor effects of DOX through the depletion of membrane cholesterol in cells, and the aim here is to evaluate the hypothesis that drug-loaded hollow microparticles with *MCD* would achieve better tumor shrinkage while improving cumulative release. Here, sample F3 was chosen for further development, with varying amounts of MCD (29.4, 58.8 or 88.2 mg). The corresponding EE of these DOX/MCD-PTX microparticles is summarized in Table 1 (i.e. samples F5 to F7).

**Figure 3:**
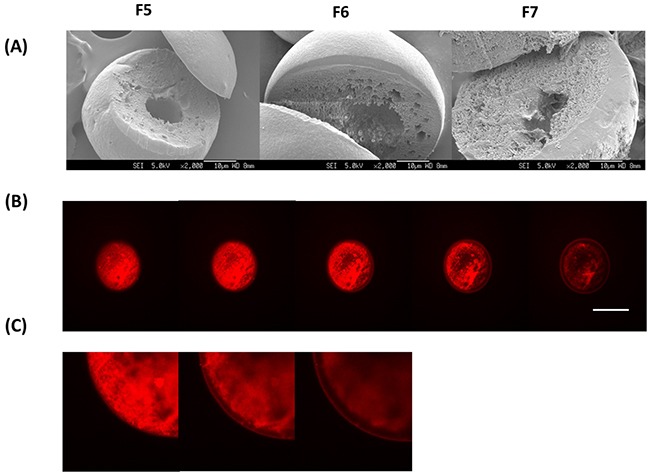
**(A)** SEM images of MCD-incorporated microparticle (F5-F7). **(B)** z-stack comprising five confocal sections was obtained for DOX (red) of F6. Scale bar = 30 μm. **(C)** z-stack comprising three zoomed-in confcoal sections of F6.

Figure 3A shows the SEM images of F5, F6 and F7. The MCD-containing microparticles were similarly spherical in shape. For these samples, the hollow cavity was less well-defined and the cross-sectioned of these microparticles showed a more porous internal structure [26]. With the addition of MCD, the size of the microparticles increased slightly – ∼45 μm (∼ 117 %) for F5 and F6 and ∼60 μm (∼ 160 %) for F7. The inclusion of MCD into the formulation however dramatically increased the EE of DOX by up to 1.6 fold (Table 1). Although DOX is a hydrophilic drug, its water solubility is limited at 50 mM. Here, the DOX/MCD complex increased the water solubility of DOX thus promoting EE of up to an average of ∼64%. In fact, from the CLSM images (Figure 3B and 3C), the red fluorescence of DOX was now observed to be more evenly distributed within the microparticle. Interestingly, achieving a higher EE for DOX was not at the expense of PTX for F5 and F6, although F7 exhibited a lower EE of PTX (70.1 ± 6.6 %). Microparticles with high MCD content tend to generate a more porous structure, and this promotes the diffusion of PTX into the aqueous phase during the evaporation process during particle fabrication [26]. An optimal MCD content is therefore required to maximize EE for both DOX and PTX.

**Figure 4:**
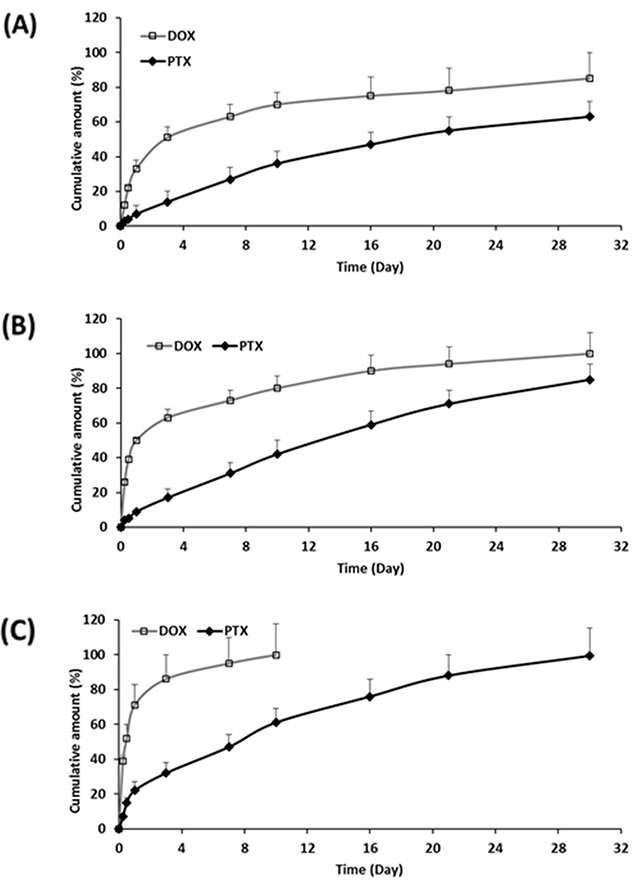
Cumulative release of DOX and PTX from **(A)** F5, **(B)** F6, and **(C)** F7 up to 30 days (n=3, mean ± S.D).

Release profiles from MCD-PLGA hollow microparticles are shown in Figure 4. The release kinetics of both drugs are summarized in Table 2, and their cumulative release plot against square-root of time is shown in Supplementary Figure 3. In these MCD-loaded hollow microparticles, both drugs were observed to have a positive correlation between release rates and MCD content, whereby a higher MCD will translate to a more rapid release. The release rate of DOX accelerated with the addition of MCD (Table 2), and displayed higher cumulative release amounts of DOX (78.1, 90.8 and 100 % at day 21, for F5, F6 and F7 respectively) (Figure 4). In addition, the cumulative released amount of PTX also increased (57.2, 73.5 or 79.4 % at day 21) with the amount of MCD. These faster release rates can be explained by the more porous structures of MCD-incorporated microparticles. The inclusion of MCD raised the hydrophilicity of the particles that promote water uptake, polymer hydrolysis (Supplementary Figure 2B) and thus drug diffusion.

### Effects of dual-drugs-loaded microparticles on tumor spheroids

Two-dimensional (2D) cell monolayers are widely used to determine cytotoxicity of drugs for up to 72 h [27]. However, 2D cell cultures often poorly mimic the micro-environment of malignant tissues, as the latter is often a more complex environment [28]. On the other hand, 3D cell culture is known to be a better representative model for actual *in vivo* environment [29–32]. Besides, the multicellular structure of 3D spheroids allows for a continuous and quantitative analysis that better mimics studies in animals [33].

DOX and PTX are by far the most common chemotherapeutic agents for cancer therapy because of their excellent anti-tumor efficacy [34, 35]. In addition, many studies have demonstrated that the co-delivery of DOX and PTX exhibited significantly higher cytotoxicity as compared to the delivery of a single drug, due to their complementary mechanisms of action. For example, DOX can bind to DNA and inhibit nucleic acid synthesis [36], while PTX promotes microtubule assembly and prevents their aggregation [37, 38]. Some clinical studies have also reported that a combination of DOX and PTX promotes better tumor regression rates compared to a single drug [39, 40]. However, any burst release of these drugs could sustain systemic toxicity that leads to adverse effects [41]. In particular, toxicity is highly dependent on the interval between the drug administrations and the duration of PTX infusion [42]. In addition, the co-administration of PTX has been reported to reduce the systemic clearance of DOX [43], thus prolonging its effect resulting in higher toxicity [44]. Therefore, a sustained release of DOX and PTX combination could be a potential approach to maximize tumor regression rates.

**Figure 5:**
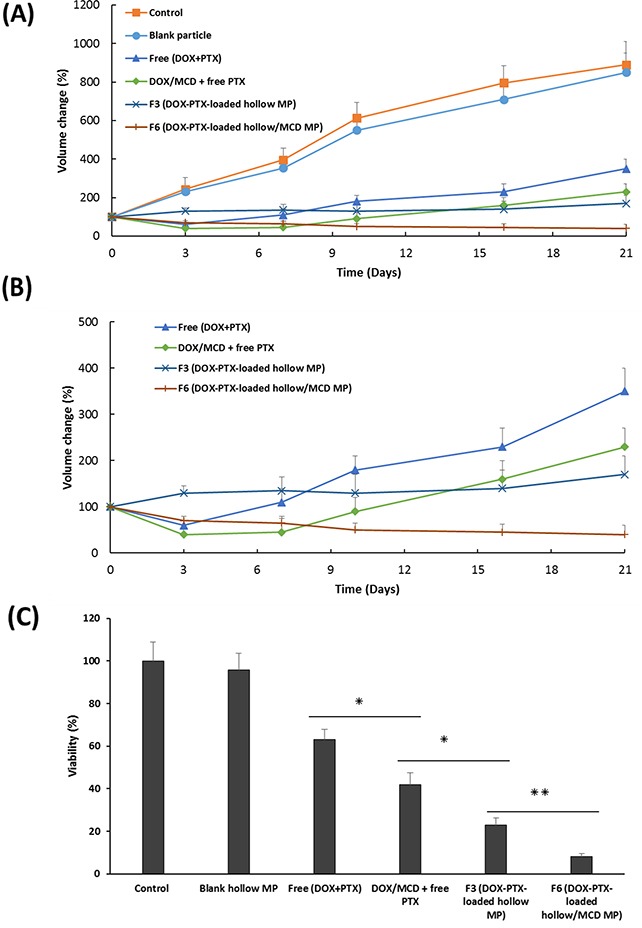
**(A)** Volume change of MCF-7 spheroids treated with blank particle, free (DOX + PTX), DOX/MCD + free PTX, F3 or F6 (at 100 ng/mL DOX and 20 ng/mL PTX) up to 21 days. **(B)** A close-up of growth curve of spheroids treated with free (DOX + PTX), DOX/MCD + free PTX, F3 or F6. **(C)** Viability of spheroids assessed by the acid phosphatase assay at the end of study (n=3, mean ±SD).

The efficacy of a combination of DOX and PTX was therefore investigated against MCF-7 spheroids for 21 days. In addition, the effects of MCD on tumor shrinkage was also studied. Comparisons were made across blank particles (drug free), free drugs (non-encapsulated drugs), F3 (without MCD) and F6 (with MCD) against control, as plotted in Figure 5. The results showed that the introduction of blank particles to MCF-7 spheroids gave the same response to that of the control. This validates the biocompatibility of the polymer used in the fabrication of these microparticles, and any cytotoxicity from the other samples has to be from the encapsulated drugs. Tumor spheroids exposed to free drugs showed a decrease in tumor volume, thus confirming the cytotoxic effects of these anticancer agents. The addition of MCD to the free drugs did have a further effect whereby a greater tumor reduction was observed. DOX/MCD is reported to be able to internalize within MCF-7 cells through the depletion of the membrane cholesterol [23]. The cells on the outer surface of spheroids are usually the first to be exposed to the cytotoxic drugs. Subsequently, apoptosis could accelerate penetration of the drugs into the primed tumors [45]. However, the effects of the drugs were worn out in due time and the spheroids continued to grow after 7 days. This recovery is due to a non-sustaining free drug exposure (6 h) to the spheroids [46]. This validates the importance of sustained release of drugs in cancer therapy. To overcome this issue, microparticles are therefore exploited to provide the sustained release of these drugs.

**Figure 6:**
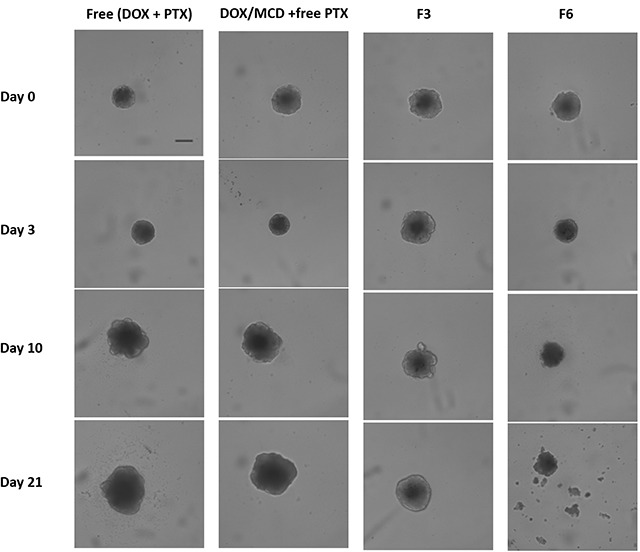
Bright-field images of MCF-7 spheroids treated with free (DOX + PTX), DOX/MCD + free PTX, F3, or F6 (at 100 ng/mL DOX and 20 ng/mL PTX) Scale bar = 200 μm.

When the drugs are encapsulated within carriers, i.e. drug-eluting microparticles, the most significant cytotoxic effects were observed. Samples F3 and F6 provided the greatest tumor regressions, whereby the volume of the spheroids at 21 days is reduced to <20% when compared to the control. This is also visually evident from the bright-field images of MCF-7 spheroids, as shown in Figure 6. Drugs encapsulated within microparticles provide controlled and sustained release of both drugs. The release of DOX ahead of PTX (Figure 4), in fact, provided an added advantage. The earlier release of DOX can sensitize the tumor to PTX that will be released at a later time point. As such, the more rapid initial release of DOX allows inhibition of cell viability in the initial stage, while the slow-release of PTX induced cell death by inhibiting microtubules disassembly [47, 48]. Drug encapsulation therefore provides the means to control how and which drugs are to be released so as to maximize on the mechanism of action of two complementary anticancer drugs. The additional combination of DOX/MCD complex was evident when comparing against F3 and F6. In particular, F6 exhibited no recovery of growth even after 21 days, and its tumor shrinkage ability is clearly evident when compared to F3 (Figure 5C and 6). The relative slow release of PTX allowed for further cytotoxicity against MCF-7 and sustaining inhibition of spheroids up to day 21. Taken together, sequential release of DOX/MCD and PTX from F6 would have the potential to further enhance therapeutic efficacy, as shown from the greater shrinkage of the tumor spheroids.

## MATERIALS AND METHODS

### Materials

Poly (D, L-lactide-co-glycolide, 50:50) (PLGA) (Intrinsic Viscosity (IV) : 1.18, Purac) and Polyvinyl alcohol (PVA) (molecular weight 30 – 70 kDa, Sigma-Aldrich) were used without further purification. DOX and PTX were purchased from Xingcheng Chempharm Co. Ltd. (Zhejiang, China) and International Laboratory (USA), respectively. Cell Counting Kit-8 (CCK-8) was purchased from Dojendo Molecular Technologies. Sodium Chloride (NaCl) and MCD were purchased from Sigma-Aldrich. PBS solution (pH 7.4) was purchased from Gibco. All other chemicals and reagents used were of analytical grade.

### Preparation of dual-drug-loaded hollow microparticles

Preparation of the hollow microcapsules encapsulating DOX and PTX was performed by double emulsion solvent evaporation method. Briefly, DOX (60 mg) and different amount of NaCl (3, 5 or 10 mg) were added in DW (0.1 mL). PLGA (0.3 g) was dissolved in DCM (5 mL). Two solutions were mixed under stirring. Subsequently, the W_1_/O emulsion was poured into 300 mL of polyvinyl alcohol (PVA) aqueous solution (5% w/v) and emulsified under overhead stirrer for 4 h at 670 *g*. The microparticles obtained were centrifuged, washed with deionized water, freeze dried and kept in -20°C for further experiments. For different size of hollow cavity, the different amount of NaCl was introduced. In order to form inclusion complex of MCD with DOX, DOX was added in deionized water (0.1 mL) with different amount of MCD.

### Drug encapsulation efficiency (EE) measurements

Microparticles (5 mg) was dissolved in DCM (1 mL). After which, deionized water (10 mL) was added and mixed using a vortex at 300 rpm (n=3). The supernatant containing hydrophilic DOX was analyzed using an ultraviolet-visible (UV-vis) spectrophotometer (Shimadzu UV-2501) at 480 nm. As for PTX, ethanol (10 mL) was added instead of deionized water to precipitate the polymers. After that, the solution was centrifuged and the supernatant was dried. Dried PTX was then dissolved in ACN for analysis. Then, the solution was analyzed using RP-HPLC with a mobile phase (65% ACN / 35% deionized water) at wavelength 227 nm. All measurements were conducted in triplicate.

### Morphological analysis

The cross-sectioned image of microparticles was taken with a scanning electron microscope (SEM, JEOL JSM-6360A). The microparticles were mounted onto a metal stub and cross-sectioned approximately at the center line using a metal blade. Then, the microparticles were coated with gold using a sputter coater (SPI-Module). The Image J software was used to measure the diameter of particle.

### Confocal laser microscopy (CLSM)

The confocal laser scanning microscope (CLSM, LSM710) was used to determine fluorescence distribution within the microparticles. The particle suspension was added to a glass slide and sealed with a cover slip. CLSM images were taken using 63×/1.40 oil objective lens and the AxioCan MRm camera (Carl Zeiss Microscopy GmbH, Oberkochen, Germany). ZEN 2012 software was used for analysis of images (Carl Zeiss, Microscopy GmbH).

### Hydrolytic degradation study

Microparticles were weighed (50 mg) and placed in glass bottles filled with PBS / 0.05 % Tween 80 (50 mL). Samples were incubated 37°C with gentle shaking. At pre-determined time points, microcapsules were collected from the bottles. Each experiment was conducted in triplicate. The molecular weight of each microcapsules was determined using the Agilent GPC 1100 using a reflective index detector (RID) with chloroform at 1 mL/min flow rate at 30°C. Molecular weights of the microparticles were calculated by the calibration curve using polystyrene standards (165-5000 kDa).

### Drug release study

*In vitro* release study was conducted in PBS (pH 7.4) with Tween 80 (0.05 %) in amber vials, and agitated using a shaking incubator at 37°C. Microparticles (5 mg) were added in PBS (5 mL). At pre-determined time points, 4ml of the release medium was remove and new medium (4 mL) was introduced to maintain sink condition. DOX concentration was analyzed with UV-vis spectrophotometer at 480 nm. PTX concentration was analyzed using RP-HPLC. In order to analyze the kinetics of drug release, the release data were fitted to Higuchi equation [49].

### Generation of MCF-7 spheroids and cytotoxicity

To generate multicellular spheroids, the MCF-7 cells were magnetically labeled using a previously established method [33, 46]. MCF-7 cells were first incubated with 750 μM BiotinSE in PBS for 30 min. The biotinylated cells were mixed with 0.025 mg/mL streptavidin paramagnetic particles and vortexed for 15s. Magnetically labeled cells at 1000 cell seeding density were dispensed into wells of 96-well round bottom low attachment plate (Corning Inc. 7007) in 100 μL medium per well. The spheroids were cultured in Dulbecco's Modified Eagle's Medium (DMEM, Gibco 11965) supplemented with 10% fetal bovine serum, 100 U mL^−1^ penicillin, and 100 μg mL^−1^ streptomycin. The spheroids were incubated in a 5% CO_2_ humidified atmosphere at 37°C.

MCF-7 spheroids (∼ 300 μm) were treated with different formulations (at 100 ng/mL DOX and 20 ng/mL PTX). Equivalent amounts of free drugs corresponding to the amounts of drugs released from the each microparticles were administered. To avoid a contact between microparticle and spheroid, the spheroids were separated from the microparticles through the use of a Transwell-96 Permeable Support with 3.0 μm pore polycarbonate membrane (Sigma CLS3385). The experiment was conducted at 37°C for 21 days. For free drug groups, drug-containing media were removed after 6 h incubation and fresh medium was replaced. Spheroid size was monitored by bright field microscopy by measuring the orthogonal diameters of each spheroid to calculate its volume.

### Acid phosphatase assay

In order to assess spheroid viability, the acid phosphatase assay was conducted at the end of experiment. The supernatant was carefully removed and replaced with 100 μL of PBS. The assay buffer (0.1M sodium acetate, 0.1% TritonX-100, supplemented with p-nitrophenyl phosphate) was added to each well at 1:1 ratio and incubated for 90 min at 37°C. Then, 10 μL of 1N sodium hydroxide (NaOH) was added. The absorbance was measured at 405 nm using a Tecan Infinite 200 microplate reader.

### Statistical analysis

Student's *t*-test was used to compare the groups. Statistically significant differences were considered when *p* value < 0.05. All data are expressed as the mean ± standard deviation from three independent experiments.

## CONCLUSION

Hydrophilic DOX and hydrophobic PTX are both encapsulated within hollow microparticles through a single-step emulsion solvent evaporation method. Through the use of NaCl as osmolyte, well-defined hollow microparticles were obtained because of the osmotic pressure achieved the use of this salt. Manipulating NaCl content changes the cavity size, shell thickness and predictably the drug release rates. MCD was next introduced to the microparticles to increase encapsulation efficiency (EE) of DOX, without compromising on the EE of PTX. When these microparticles (without and with MCD; F3 and F6 respectively) were added to 3D tumor spheroids of MCF-7 cells, a dramatic reduction in spheroid volume was observed, when compared to control, blank particles and free drugs. The addition of MCD (F6) also provided additional benefit of tumor shrinkage because of its ability to enhance the toxic effects of DOX. This study proves the hypothesis that sustained-releasing drug-loaded hollow microparticles, with *MCD*, can achieve better tumor shrinkage outcomes in 3D MCF-7 spheroids. Such a delivery system may hold great advantages in terms of manipulating release profiles of multiple drugs for future exploitations in cancer therapy.

## SUPPLEMENTARY MATERIALS FIGURES AND TABLES


