# Supplementary material for: Sustained-releasing hollow microparticles with dual-anticancer drugs elicit greater shrinkage of tumor spheroids
Source: Oncotarget. 2017 Aug 24;8(46):80841–52. doi: 10.18632/oncotarget.20591 (PMC5655243; doi:10.18632/oncotarget.20591)
Supplement: Supplementary file 1 [file oncotarget-08-80841-s001.pdf]

Sustained-releasing hollow microparticles with dual-anticancer drugs elicit greater shrinkage of tumor spheroids

SUPPLEMENTARY MATERIALS

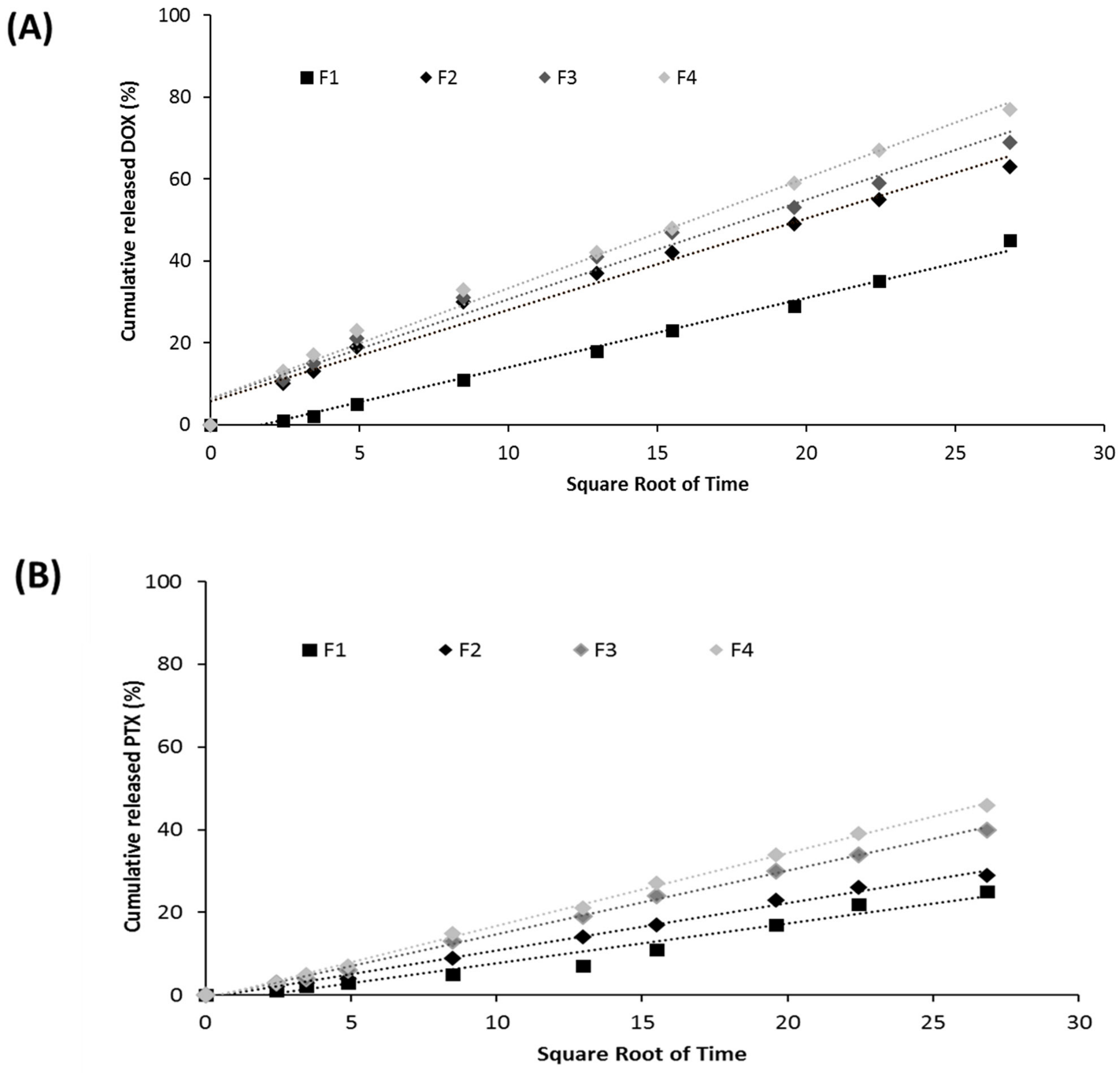

Supplementary Figure 1: *In vitro* release of **(A)** DOX and **(B)** PTX from solid (F1) and hollow (F2-F4) microparticles as a function of square root of time (n=3).

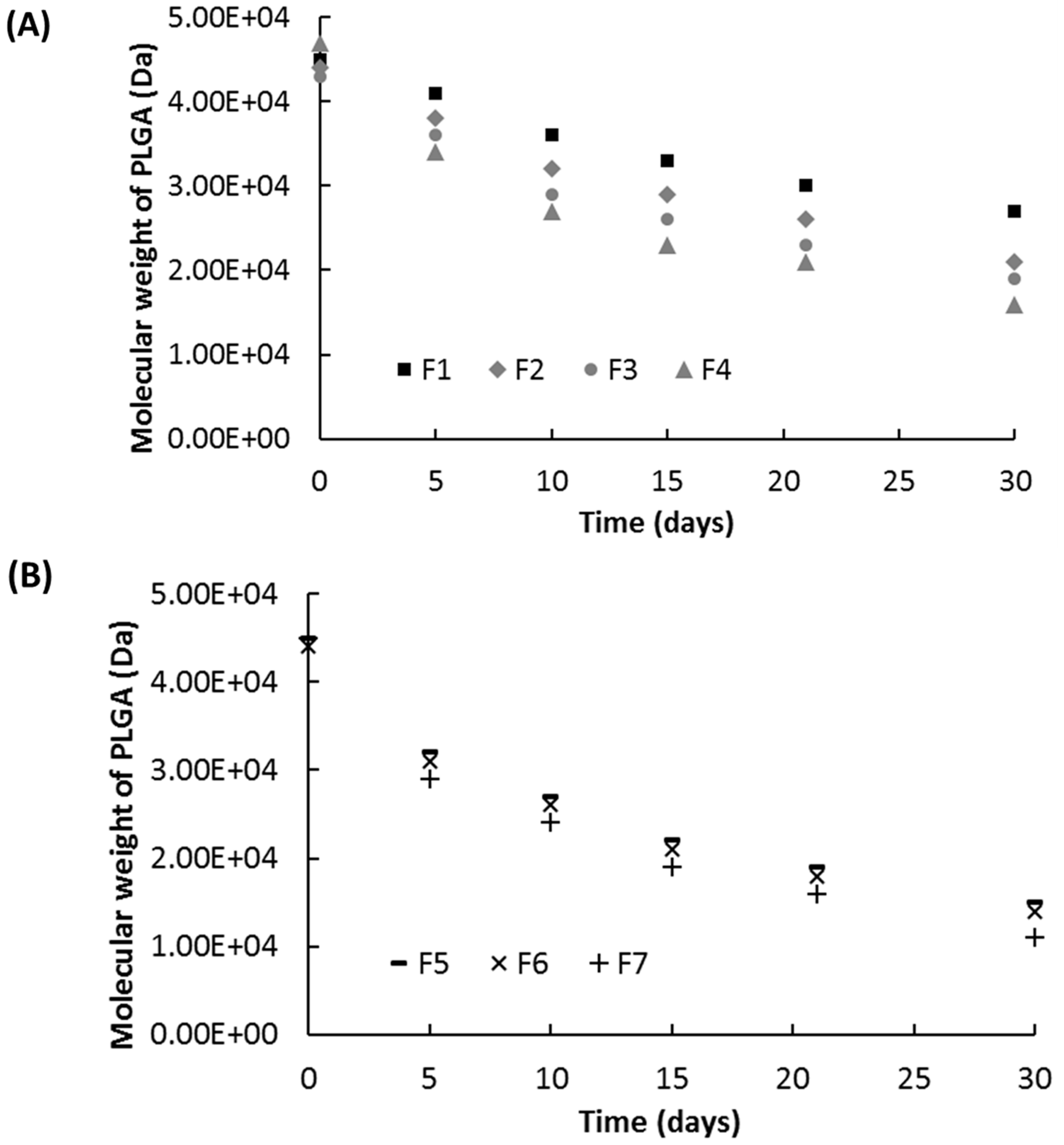

Supplementary Figure 2: Average molecular weight of PLGA of the degrading **(A)** microparticles (F1-F4) and **(B)** microparticles (F5-F7) over time (n=3).

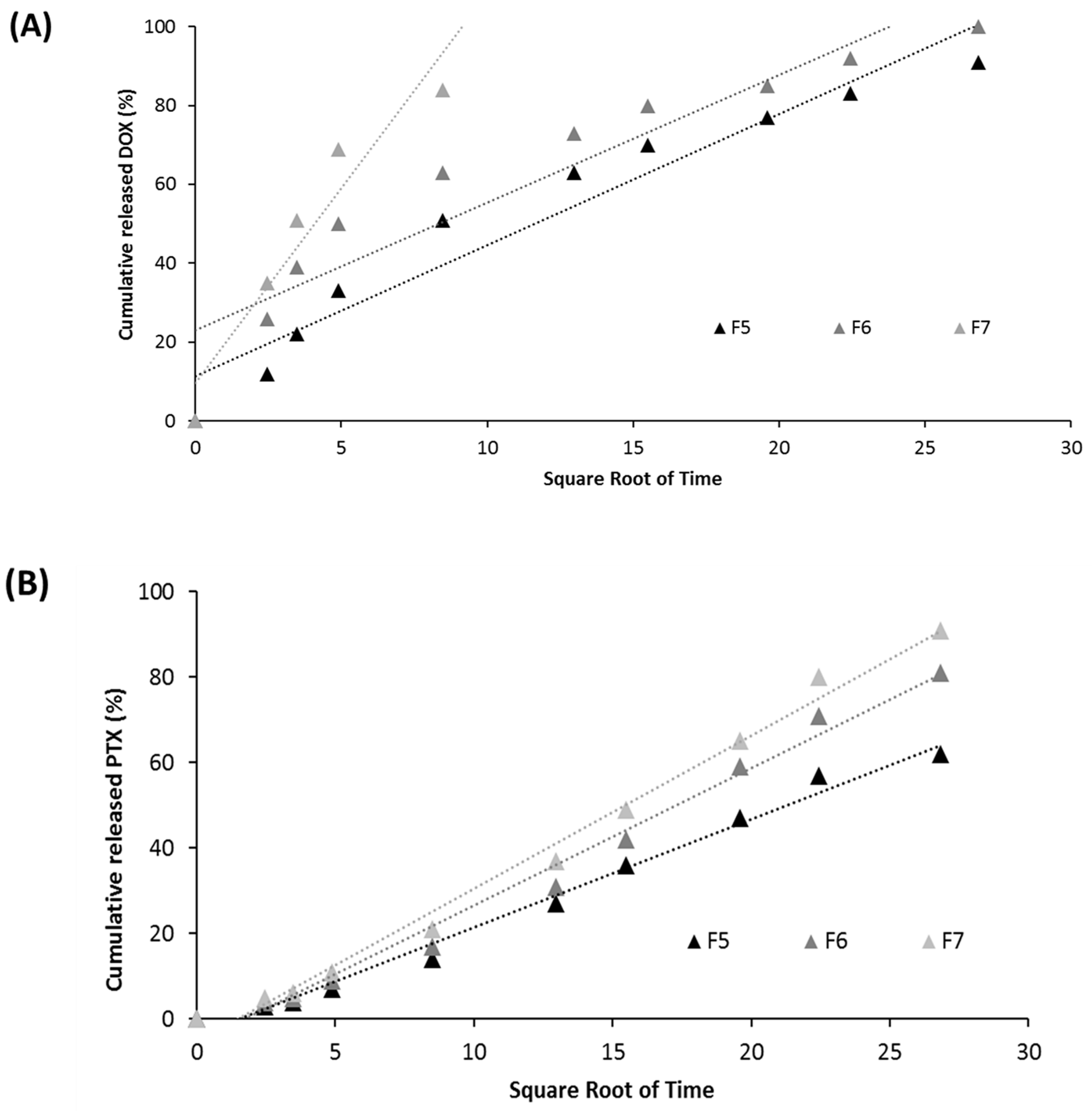

Supplementary Figure 3: *In vitro* release of **(A)** DOX and **(B)** PTX from MCD-incorporated microparticle (F5-F7) as a function of square root of time (n=3).
